# Expanding the Donor Pool to the Ultimate Level: Introducing the Revolutionary Hybrid Dual Graft Liver Transplant Using Domino and Living Donors

**DOI:** 10.1097/TXD.0000000000001681

**Published:** 2024-07-26

**Authors:** Ahmed Zidan, Hammam Momani, Bodhisatwa Sengupta, Rehab Abdullah, Razan Bader, Iftikhar Khan, Mansour Tawfeeq, Mohammed Al Qahtani

**Affiliations:** 1 Organ Transplant Center of Excellence, King Fahad Specialist Hospital, Dammam, Saudi Arabia.

## Abstract

**Background.:**

Innovative solutions are crucial as the demand for liver transplants continues to outpace available grafts. Dual graft liver transplantation offers a promising avenue to address graft volume challenges while minimizing donor risks. This report introduces a groundbreaking approach, combining a full organ domino donor graft with a living donor graft for a hybrid dual graft liver transplant.

**Brief Report.:**

A 2-y-old child with Maple syrup urine disease and a 40-y-old adult with end-stage liver disease became the focus of this unique case. A hybrid dual graft liver transplant was executed, uniting the domino donor’s full organ graft with a living donor’s left lateral segment. Precise vascular and biliary reconstructions facilitated a successful transplant.

**Conclusions.:**

The hybrid dual graft liver transplant, merging domino donor and living donor grafts, presents a viable strategy to combat graft shortages, particularly in regions predominantly reliant on living donor transplants. Despite challenges, this pioneering approach should be embraced by established liver transplant centers because it enables concurrent living donor liver transplantation while prioritizing donor safety and recipient outcomes.

The demand for liver transplants (LTs) has constantly been on the rise, leading to the development of innovative solutions to overcome the shortage of grafts. One such solution is the dual graft LT, where 2 separate liver grafts are transplanted into a single recipient.^1,2^ In addition to providing the required volume, it mitigates the donor risk with minimization of the resected liver volume from the donor that has been found to correlate with posthepatectomy mortality rates; worldwide donor mortality estimates for left lateral segmentectomy is 0.1%, whereas for right lobe donors is 0.4%–0.5%.^3^ Moreover, the cultural obstacles in certain countries such as the Kingdom of Saudi Arabia have limited the implementation of deceased donor LTs, resulting in a scarcity of liver grafts.

Maple syrup urine disease (MSUD) is an autosomal recessive disorder caused by mutations in the branched-chain ketoacid dehydrogenase complex, leading to the accumulation of branched-chain amino acids. Symptoms include neurological disabilities, coma, and mortality. Although dietary management helps, severe cases may require LT to prevent complications. LT is crucial as the liver plays a significant role in branched-chain ketoacid dehydrogenase production. Donor livers from patients with MSUD are viable for domino LT (DLT), expanding the donor pool. Recipients of MSUD donor livers are spared from the disease, as branched-chain amino acid metabolism mainly occurs in extrahepatic tissues. DLT thus offers a strategic solution as a source of liver grafts. We present the first-ever reported case in the literature of prescribed dual graft LT using hybrid living donor and domino donor grafts.^4^

## CASE REPORT

A 2-y-old child, weighing 13 kg, was diagnosed with MSUD with multiple metabolic crisis that required dialysis multiple times. Her Mother stepped forward to be a living liver donor for her. Unfortunately, there were no suitable recipients on the pediatric waiting list with the same blood group for this MSUD liver as DLT. However, on the adult waiting list, there was a 40-y-old man, weighing 80 kg, diagnosed with alcohol-induced end-stage liver disease, with marked uncontrolled ascites as a sign of decompensation with an Model of End Stage Liver Disease score of 26. He had been on the waiting list for 1 y without any available living donors and no available deceased donor livers at this time. Given that the child’s liver would not suffice for the adult recipient’s needs, and the risk of developing “small-for-size syndrome” was high, we decided to perform a hybrid dual graft LT by combining the full liver graft from the MSUD child with the left lateral segment from an altruistic living donor, to meet the metabolic demands of the adult recipient and reduce the risk for the donor.

Our hospital has an altruistic donor registry, allowing individuals to donate to any potential recipient anonymously. We informed and counseled an altruistic donor, who was admitted and prepared for the procedure.

The procurement of the MSUD liver went smoothly, and living donor LT (LDLT) from the Mother to the patient with MSUD was uneventful. Then, the left lateral segmentectomy from the altruistic donor was performed with an uneventful event, with an operative time of 360 min (Table 1).

**TABLE 1.** Demographic and operative data of both donors and the recipient

Donor 1 operative data	Donor 2 operative data
MSUD donor full graft CIT 400 min Full graft with standard anatomy Graft weight 468 g	LLS OR time 360 min WIT 38 min Blood loss 50 mL LLS (1 HV, 1 PV, 1 HA, 1 BD) Graft weight 209 g
**Recipient operative data**	
Full domino graft on the right side and LLS on the left side OR time 755 min Blood loss 5 L Total liver weight 677 g (GRWR 0.84)	
**First graft**	**Second graft**
3 HVs as 1 stoma after venoplasty to RHV with IVC extension of the patient MPV of the graft to RPV of the patient. CHA of the graft to RHA of the patient CBD of the graft to CBD of the patient	LHV of the graft to LHV of the patient LPV of the graft to LPV of the patient LHA of the graft to LHA of the patient 1 BD of the graft to the jejunum

BD, bile duct; CBD, common bile duct; CHA, common hepatic artery; CIT, cold ischemia time; GRWR, graft to recipient weight ratio; HA, hepatic artery; HV, hepatic vein; IVC, inferior vena Cava; LHA, left hepatic artery; LHV, left hepatic vein; LLS, left lateral segment; LPV, left portal vein; MSUD, Maple syrup urine disease; MPV, main portal vein; OR, operation; PV, portal vein; RHA, right hepatic artery; RHV, right hepatic vein; RPV, right portal vein; WIT, warm ischemia time.

The full organ domino donor graft weighed 468 g, and the left lateral segment weighed 209 g, making the total liver volume 677 g with a graft weight to recipient weight ratio of 0.84.

### Operative Procedure

**FIGURE 1.**
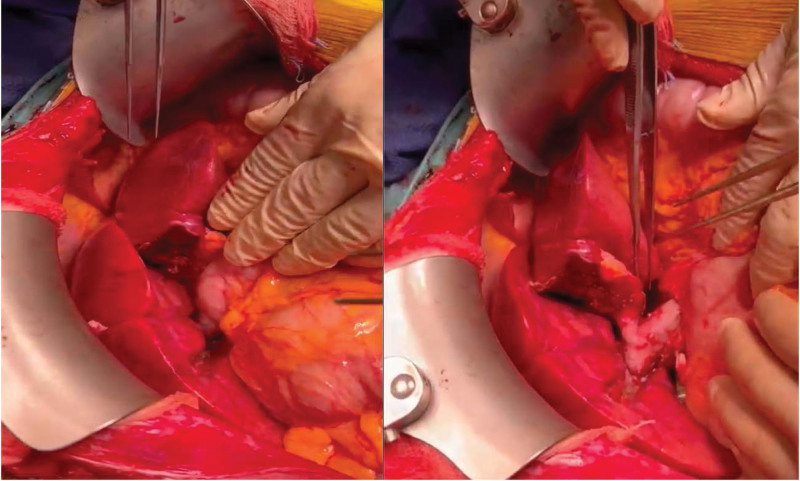
Intraoperative images showing both liver grafts.

In the recipient operation, hepatectomy was performed with a piggyback technique. Following this, we implanted the domino donor full organ graft on the right side of the patient by anastomosing the 3 hepatic veins as single anastomosis after venoplasty to connect all of them together, creating 1 stoma, to the right hepatic vein opening in the recipient. This was achieved using the triangular technique without the use of any patches. We then connected the main portal vein of the graft to the right portal vein of the recipient. The second graft, which was the left lateral living donor graft, was implanted in the left side of the recipient. This was achieved by anastomosing the left hepatic vein of the graft with the left hepatic vein opening, followed by the left portal vein of the graft with the left portal vein of the recipient. Then, after reperfusion of the second graft, we started arterial reconstruction by anastomosing the common hepatic artery of the domino graft to the right hepatic artery of the recipient and anastomosing the left hepatic artery of the graft to the left hepatic artery in the recipient (Figure 1).

Biliary reconstruction of both grafts was then attained. The full organ domino graft on the right side was reconstructed with duct-to-duct anastomosis with the recipient common bile duct, whereas the left lateral segment graft, which had 1 bile duct, was anastomosed to the Roux limb hepaticojejunostomy.

**FIGURE 2.**
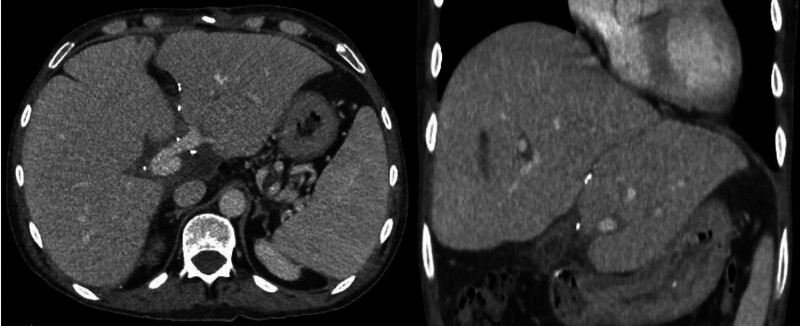
Computed tomography after 1 mo of transplant.

The estimated operative time was 755 min, with a blood loss of 5 L; no cell saver was used and a total of 11 units of packed red blood cells, 12 units of fresh frozen plasma, and 6 units of cryoprecipitate were given. The postoperative monitoring (Table 2) found the patient’s course to be smooth and uneventful except for a chylous leak, which was managed conservatively and resolved spontaneously, with the patient being discharged on postoperative day 28. Subsequent follow-up ultrasonography and computed tomography showed patent vessels in both liver grafts (Figure 2).

**TABLE 2.** Table showing liver and renal parameters in the first 90 d after transplant

	D1	D7	D10	D30	D60	D90
INR	3.2	1.8	1.2	1	1.1	1.0
Bilirubin, µmol/L	134	162	127	23	12.5	10.4
Creatinine, µmol/L	51	59	55	58	80	72
AST/ALT, U/L	451/709	81/162	117/123	57/118	23/43	25/43

ALT, alanine Transaminases; AST, aspartate transaminases; INR, international normalized ratio.

## DISCUSSION

The use of deceased donor LT is not well established in many Middle Eastern countries, including the Kingdom of Saudi Arabia. This shortage of potential liver donors provokes us to find alternative innovative solutions, particularly with the growing gap between the demand for LTs and the available donor pool. As a result, LDLT remains the primary option for most patients with life-threatening liver disease.^2^ DLT for patients with MSUD not only represents a significant advancement in the personalized treatment of metabolic disorders but also serves as a strategic solution to enhance the efficiency and accessibility of LT services on a broader scale.^5^

In our center, we rely mainly on LDLT as the primary option for LT patients. The good outcome on both donor and recipient sides motivated more altruistic donors to register for liver donation. Moreover, we have started our DLT program in 2016, with almost 50% of DLTs in Saudi Arabia performed in our center.

The minimum graft size should be at least 40% of standard liver volume to optimize recipient outcomes or produce a graft to recipient weight ratio of ≥0.8. This ensures that the graft volume is sufficient to meet the metabolic demands of the recipient and prevents small-for-size syndrome.^3^

One of the most important surgical innovations in LT is the dual graft LT, which addresses the imbalance between recipient liver volume requirements and the potential donor’s liver volume.^1^ All dual graft LTs in current literature involve combining 2 living donor liver grafts to achieve an acceptable graft volume for the recipient.^2,6^ However, in this case report, we combined an MSUD donor full organ liver graft as a DLT with a living donor liver graft for the first time ever. We have used many surgical innovations in the field of LT to obtain the maximum benefit for the recipient. We combined dual graft LT, altruistic liver donation, and DLT, resulting in this unique hybrid domino donor and living donor dual graft LT.

The Domino full organ graft was implanted on the right side and a biliary system reconstructed as a duct-to-duct anastomosis. On the left side, the living donor liver was implanted with jejunal Roux limb anastomosis because it allowed for easier flipping upward and to the left to obtain greater exposure because it is easier to flip it upward and to the left to have more exposure of bile ducts, which may be more difficult in the full organ liver graft. This flipping of the graft may require more distance, which could be achieved by jejunal Roux anastomosis.

In conclusion, we consider hybrid dual graft domino donor and LDLT a feasible way to expand the donor pool in countries with low access to deceased donor LT and to properly use the domino donor grafts, especially in countries that depend mainly on living donors with limited access to deceased donors as in the case in the Kingdom of Saudi Arabia. Despite of its technical and logistical challenges, hybrid dual graft LT should be assimilated into the armamentarium of a mature LT center because it has been shown to facilitate the completion of an LDLT and DLT while jointly prioritizing safety in the individual donor.
